# Macro-aspartate Aminotransferase: Misleading Finding in a Patient with Non-alcoholic Fatty Liver Disease

**DOI:** 10.7759/cureus.5042

**Published:** 2019-06-29

**Authors:** Neha Sharma, Umar Darr, Amir Darr, Gagan K Sood

**Affiliations:** 1 Abdominal and Liver Transplantation, Baylor College of Medicine, Houston, USA; 2 Science, University of Limerick, Limerick, IRL; 3 Abdominal Transplantation, Baylor College of Medicine, Houston, USA

**Keywords:** macroast, non-alcoholic fatty liver disease, macroenzymes, immune dysregulation, peg precipitation

## Abstract

Persistent elevation of aspartate aminotransferase (AST) activity in serum due to the presence of a macro-enzyme form of AST (macro-AST) may lead to diagnostic confusion in many clinical conditions, particularly in those associated with chronic liver disease. We present a case of macro-AST in a patient with non-alcoholic fatty liver disease in which polyethylene glycol precipitation confirmed the cause of disproportionately elevated AST as macro-AST.

## Introduction

Macro-enzymes are high molecular mass complexes of plasma enzymes with immunoglobulins (e.g., IgG, IgA or IgM) or other plasma components [1]. They are formed as a result of immune dysregulation. Their reduced plasma clearance and prolonged half-life results in elevated serum activities due to the “trapping” of enzymes in serum and reflect as unusually high values on laboratory investigations without a plausible explanation.

Isolated elevation of aspartate aminotransferase (AST) due to macro-enzyme-AST (macro-AST) is a benign and rare condition [2]. Because of its rarity, macro-enzyme testing is not routinely performed in clinical practice. Therefore, the diagnosis is often delayed with a prolonged work-up and may include invasive evaluations like a liver biopsy. We report a case of isolated AST elevation with non-alcoholic fatty liver disease due to the presence of macro-AST.

## Case presentation

**Figure 1:**
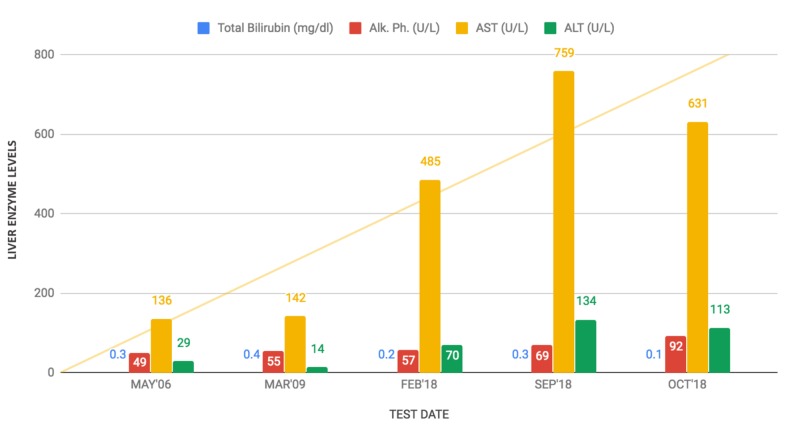
Persistent and disproportionate elevation of AST compared to ALT for over a decade. Enzyme (reference range): Total bilirubin (0.1-1.0 mg/dL) Alk. Ph.: alkaline phosphatase (30-110 U/L) AST: aspartate aminotransferase (0-45 U/L) ALT: alanine aminotransferase (0-55 U/L)

A 46-year-old African-American woman was referred to our hepatology clinic for evaluation of abnormal liver enzymes detected on routine blood work. The elevated AST level of 136 U/L was first noted in 2006, and other liver enzymes were within reference ranges. Subsequent measurements showed an upward trend for persistent AST elevation, as shown in Figure 1.

The physical examination was unremarkable, and the patient had no stigmata of liver disease. A review of her laboratory data revealed disproportionately elevated AST levels. A comprehensive work-up for elevated AST including screening for hemolysis, acute and chronic viral hepatitis, autoimmune hepatitis, alpha-1 antitrypsin, ferritin levels, creatine kinase (CK), and aldolase was unrevealing.

**Figure 2:**
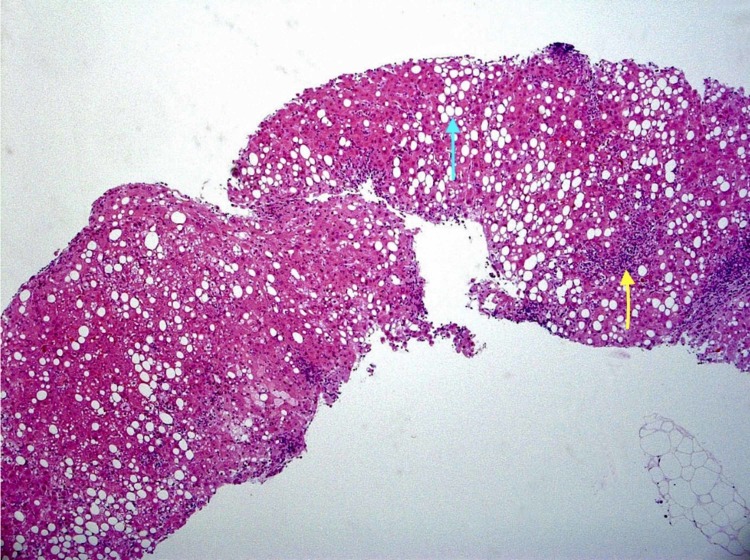
Liver biopsy showing steatohepatitis (yellow arrow), steatosis, moderate ballooning (blue arrow). No fibrosis or iron stores seen.

An ultrasound and computed tomography (CT) scan revealed a normal liver, spleen, and pancreas on two separate occasions, with one year between the scans. Liver biopsy findings were suggestive of steatohepatitis, as shown in Figure 2.

As the tests did not reveal the cause of the unusually high AST levels, macro-AST was suspected. To confirm this, a blood sample was tested for macro-AST by the polyethylene glycol (PEG) precipitation method. Serum AST activity before precipitation was 808 U/L, but it dropped to 24 U/L post-PEG precipitation. The results were consistent with the presence of macro-AST (i.e., 97% of the activity is precipitated with PEG), confirming our diagnosis.

## Discussion

Several enzymes (e.g., amylase, CK, lactate dehydrogenase, and AST) complexed with immunoglobulins have been described in the literature as macro-enzymes, with macro-amylasemia being the most frequently reported. The incidence of immunoglobulin-complexed AST has not yet been established in the general population, but it appears low because the biochemical methods for diagnosis are not readily available in the laboratory. 

Elevated liver enzymes, such as aspartate aminotransferase and alanine aminotransferase, most commonly reflect hepatocellular injury, and therefore, prompt extensive serological, radiological, and sometimes histological evaluations are required for assessment of liver disease. The most common conditions to be ruled out include alcoholic hepatitis, viral hepatitis, hemochromatosis, autoimmune hepatitis, Wilson's disease, alpha-1 antitrypsin deficiency, medication use and congestive hepatopathy. The patient should be tested for hepatitis B and C, serum iron studies, autoantibodies, serum ceruloplasmin and serum alpha-1 antitrypsin levels. When no liver-related etiology is found, attention should be diverted to extra-hepatic sources of injury such as rhabdomyolysis and hemolysis, and the patient should be tested for myoglobinuria and peripheral blood smear study and serum haptoglobin levels. An isolated or disproportionate elevation in AST in the absence of these processes warrants an evaluation of macro-AST [3].

Macro-AST is typically a diagnosis of exclusion. The clinician should be aware of the possibility of this diagnosis and retain a high index of suspicion for this condition. Thorough patient history and routine laboratory tests are important to ensure that no associated or coexisting condition explains the elevated AST levels. The next step is confirmatory diagnostic testing such as assessing protein electrophoresis, precipitation with PEG, exclusion gel filtration chromatography, or activation assays with pyridoxal-5-phosphate [4-6].

We found no discernible etiology for macro-AST in our patient. Therefore, we concluded that it was a benign elevation of macro-AST that manifested incidentally. The treatment involved reassurance with routine monitoring of liver enzymes.

## Conclusions

This case illustrates the need to consider macro-AST as a cause of persistent or disproportionate elevation of AST in a patient with non-alcoholic steatohepatitis. The use of PEG is a simple and effective method for detecting macro-enzymes; it is a highly sensitive and specific test but is not widely available. This report emphasizes the need for a high clinical index of suspicion and early testing for macro-AST to avoid unnecessary expensive and invasive investigations. The presence of macro-AST is not a transient phenomenon, and elevated laboratory values attributable to immunocomplex enzymes may persist for many years. Therefore, a thorough documentation in patient notes is vital to avoid future diagnostic dilemmas. No medication is indicated, and reassurance is the mainstay of treatment.
